# Case of Distention of the Unimpregnated Uterus with Blood

**Published:** 1827-12

**Authors:** John Paul

**Affiliations:** Member of the Royal College of Surgeons in London.


### PATHOLOGY OF THE UTERUS.

### *Case of Distention of the unimpregnated Uterus with Blood.* By John Paul, M.D. Member of the Royal College of Surgeons in London.

The following case, I hope, will not be deemed unworthy of record in the London Medical and Physical Journal, as it furnishes us with a valuable pathological fact: for, so far as I know, distention of the unimpregnated uterus with blood to the same extent, and occurring in the same manner, has not hitherto been taken notice of. If in this I am mistaken, want of reference, to which a provincial surgeon is necessarily subjected, must plead my excuse.

A. B—, an unmarried lady, ætat. forty-three, delicate in her health and of lax fibre, has been three times the subject of the complaint, of which I am now to attempt a short description. She had the first attack in the autumn of 1820, the second in the summer of 1822, and the third in December 1826. Previous to this last illness, she had undergone great fatigue in attending a near relative, who was very ill of fever, and in whose recovery she was deeply and anxiously interested. For many nights during her attendance she had not been in bed, and was much exposed to cold. Her menstrual discharge came on whilst she was thus engaged, and, after continuing nine days, greatly too profuse, it suddenly stopped; and on the following day her abdomen was tumid. She was now obliged to confine herself to bed.

The discharge returned two days after; and, when I saw her on the 8th of December, she had been five days confined to bed, during which time she had had no sleep, and complained that she was distressed with headache and great uneasiness in her back. She was extremely despondent, and exhibited occasionally symptoms of hysteria; pulse was ninety-eight; heat of skin was rather above the natural standard. On examining the abdomen, it was with ease ascertained that the uterus was greatly enlarged, its fundus extending very near to the umbilicus; and in this there could be no deception, as the abdominal parietes were so thin that the outlines of the distended uterus could be distinctly traced. It was fully as large as it is at the completion of the fifth month of utero-gestation. When the patient lay on her back, the medial line intersected the tumor exactly; when she lay on her side, the tumor inclined to that side.

The history of the two preceding attacks, as I shall presently show, proved beyond all doubt that this enlargement was from distention of the uterus with blood. It was proposed, in the first place, to irritate with the finger the os uteri, with the view of bringing on uterine contraction; but the patient could by no entreaty be prevailed with to submit to this, and, consequently, no examination per vaginam could be obtained. Sleep was procured by the exhibition of an opiate, and in the course of a few days, the discharge was entirely arrested by the administration of the super-acetate of lead, combined with small quantities of opium. To give support to the abdomen, a roller was applied tightly round the body.

On the 25th January, 1827, the menstrual discharge reappeared, and continued four days. It was thick and of a dark colour, but was not fœtid, nor did it contain any clots. By this discharge, the tumor was one-half reduced in size; and, on the return of menstruation at the usual period, the whole of the blood retained in utero was thrown off, rather in a fœtid state; when, on the most minute examination, no appearance of tumor was perceptible above the pubes. Since that time there has been no return of the complaint.

This patient, preceding the first attack in 1820, was bled from the arm on account of pain in her side, and on this hemorrhage from the uterus immediately supervened: the discharge externally was arrested by the application of cold cloths, but blood continued to be poured out internally, as the uterus was soon so much distended as to be in contact with the umbilicus. Anasarca, from the loss of blood, was now general, and the patient appeared to be in a very precarious state. About three months afterwards, menstruation came on, and a good deal of clotted blood was discharged; but the swelling did not entirely disappear till after the third menstrual period. After this her health was soon restored.

It was immediately after menstruation was suddenly suppressed by exposure to cold, whilst it was profuse, that this distention of the uterus occurred the second time. At the end of two months, menstruation again made its appearance, when a quantity of dark-coloured blood, in a very fœtid state, was suddenly discharged; and the patient nearly lost her life on account of the profuseness of the discharge, and the distance at which she lived from medical assistance.

There was now no remains of uterine enlargement; but, such was the state of weakness to which she was reduced, along with the anasarcous swellings, which had again supervened, that the re-establishment of her health required great care and a considerable length of time.

In the treatment of this case, there was nothing remarkable, and it is therefore unnecessary to give any detail of it. It was conducted on the principle of preventing, as much as possible, the loss of blood whilst the uterus was in a state of distention; and, after the retained blood was discharged, every means were had recourse to that could give tone to the system. During the distention, nothing was tried with the view of bringing on uterine contraction, except friction and the use of a bandage. For that purpose I proposed to irritate the os uteri with my finger, but it was decidedly objected to. It did also occur to me to try the Secale Cornutum during the last attack, but none could be procured in this part of the country.

When we reflect on the anatomical structure of the uterus, it is difficult to conceive the possibility of its distention in the manner now detailed; but the facts adduced incontrovertibly prove, in my opinion, that such was the case. As the distention supervened immediately on the sudden obstruction of the menses from the effect of cold, the os uteri, I think, must have been firmly closed either by spasm or a clot of blood, or probably by both. Its mouth being thus shut, the uterus, it would appear, became dilated from the impulse of the internal hemorrhage.

*Elgin; 16th October*, 1827.

---

TRANSFUSION.

